# Microchimerism as Post-Transplant Marker of a Chronic Rejection Process

**DOI:** 10.3390/ijms241310603

**Published:** 2023-06-25

**Authors:** Jerzy Sieńko, Maciej Kotowski, Wiktoria Czarnecka, Albert Podkówka, Karol Tejchman, Katarzyna Kotfis, Samir Zeair, Zenon Czajkowski, Karolina Skonieczna-Żydecka

**Affiliations:** 1 Institute of Physical Culture Sciences, University of Szczecin, 70-453 Szczecin, Poland; jsien@poczta.onet.pl; 2 Department of General Surgery and Transplantology, Pomeranian Medical University in Szczecin, 70-111 Szczecin, Poland; 3 Scientific Circle at Department of Biochemical Sciences, Pomeranian Medical University in Szczecin, 71-460 Szczecin, Poland; 4 Department of Anesthesiology, Intensive Therapy and Acute Intoxications, Pomeranian Medical University in Szczecin, 70-111 Szczecin, Poland; 5 General and Transplant Surgery Ward with Sub-Departments of Pomeranian Regional Hospital in Szczecin, 71-455 Szczecin, Poland; 6 Department of Intensive Care, Pomeranian Regional Hospital in Szczecin, 71-455 Szczecin, Poland; 7 Department of Biochemical Sciences, Pomeranian Medical University in Szczecin, 71-460 Szczecin, Poland

**Keywords:** microchimerism, renal transplantation, rejection

## Abstract

The risk of losing a transplanted organ is high, and non-invasive markers to warn of this phenomenon are still being sought. We investigated the impact of post-transplant microchimerism on the function of the transplanted kidney. The study included 100 kidney transplant recipients, mostly women. All transplanted organs were from opposite-sex deceased donors. Microchimerism was assessed using multiplex PCR. Male DNA was detected in all urine samples from female recipients and in 13/56 blood samples from female kidney recipients. Female DNA was found in 31/44 urine samples from male recipients, but in none of the blood samples. Microchimerism in the urine of female recipients correlated positively with blood urea (Rs = 0.45; *p* = 5.84 × 10^−4^) and K^+^ ions (Rs = 0.29; *p* = 0.03), while microchimerism in the blood of female recipients also correlated positively with blood urea (Rs = 0. 28; *p* = 0.04), cystatin C (Rs = 0.31; *p* = 0.02) and the number of incompatible HLA alleles (Rs = 0.42; *p* = 0.01). A history of DGF was associated with higher urinary donor DNA concentrations in female recipients.: Post-transplant microchimerism may serve as a potential marker of chronic kidney rejection.

## 1. Introduction

Kidney transplantation is widely recognized as the most effective treatment for chronic kidney failure, offering not only the highest survival rate compared with other treatments, but also the highest level of patient comfort [1,2]. In Poland, the number of kidney transplants performed in 2021 was 753 [3], with the annual survival rate of patients after kidney transplantation fluctuating around 95%

Many years of experience of specialists and continuous intensive development of knowledge of possible immunosuppressive treatment methods result in a yearly decrease in the number of acute rejection episodes and an increase in the number of functioning organs in the first post-transplantation period. Unfortunately, modern transplantation medicine is still struggling with the problem of chronic rejection, which leads to dysfunction and ultimately organ loss [4]. According to available data, 90–95% of kidneys function properly after the first year following transplantation, and after 10 years, only one in two living donor kidneys and one in three deceased donor kidneys are still functioning [5]. As a result, researchers are constantly looking for ways to slow or stop chronic graft rejection. At the same time, the search is on for non-invasive markers that would warn of this process and possibly illustrate its dynamics. One such marker could be quantified post-transplant microchimerism.

Microchimerism refers to the coexistence of two genetically distinct populations in an organism, one of which is present in very small numbers. The simplest mechanism for this phenomenon in the human body is the mixing of blood from two organisms, the best example being maternal–fetal microchimerism. A woman has been shown to have cells from previous pregnancies in addition to her own genetic material [6,7,8]. Men, on the other hand, retain traces of maternal blood in their bodies [6,7,8]. Another source of microchimerism may be hematopoietic cell and organ transplants and transfusions of blood products [9].

Microchimerism appears to be particularly important in the context of transplantation. Donor DNA in the recipient’s organism originates from damaged cells of the transplanted organ. The source of damage can be the cytotoxic effects of immunosuppressive drugs, acute infection and the process of acute or chronic rejection. DNA from dying cells is broken down into nucleosomes and oligomers and then mostly recycled. However, some DNA does not undergo phagocytosis and ends up in the blood. Importantly, the amount of DNA in circulation correlates with the number of dying cells. The renal barrier partially transmits polymeric DNA from apoptotic cells in sufficient quantities to be detected by genetic analysis techniques such as PCR and hybridization [10]. The natural source of kidney donor cells is the sediment from the recipient’s urine. Damaged cells or cells that die in the process of apoptosis are ‘swept up’ by the urine that forms and is excreted from the body.

The role of microchimerism in the recipient’s organism remains controversial, as scientists are still unable to agree whether it contributes to immunological tolerance or activates the process of chronic rejection. However, a clear answer to this question could make the quantification of microchimerism a useful marker for assessing the success and risks of transplantation. This could lead to the replacement of an invasive procedure such as organ biopsy with a non-invasive and patient-friendly test. The present study is an attempt to determine the impact of microchimerism on the function of the transplanted kidney based on biochemical analytes measured at the time of microchimerism detection. In addition, these indices were evaluated in relation to the state of the recipient’s immune system by determining selected immunocompetent cells in the blood: T lymphocytes—CD8+ and CD4+, NK cells, B lymphocytes, monocytes and CD4+ CD25+ regulatory cells. To exclude the possible influence of the presence of maternal–fetal microchimerism and the patient’s previous blood transfusion on the results obtained, a comparison was made of the microchimerism levels in recipients with and without PRBC and in women with and without male offspring.

## 2. Results

### 2.1. Characteristics of the Study Group

The study group included 100 patients aged 20–78 years with a mean age of 49.91 years, 56% of whom were women (n = 56). The mean follow-up was 1817 days after surgery. Body mass index ranged from 18 to 44 kg/m^2^ (mean 26.41). Detailed characteristics of the study group are shown in Table 1.

The main causes of renal failure in the study group were glomerulonephritis (36.63%), ADPKD (12.87%), reflux (11.88%), arterial hypertension (10.89%), diabetes (7.92%), IGA nephropathy (1.98%) and urolithiasis (1.98%). Other causes were found in 15.84% of patients.

### 2.2. Microchimerism in Blood and Urine of Female Kidney Recipients from Male Donors

#### 2.2.1. Presence of Male DNA in Female Urine

**Figure 1:**
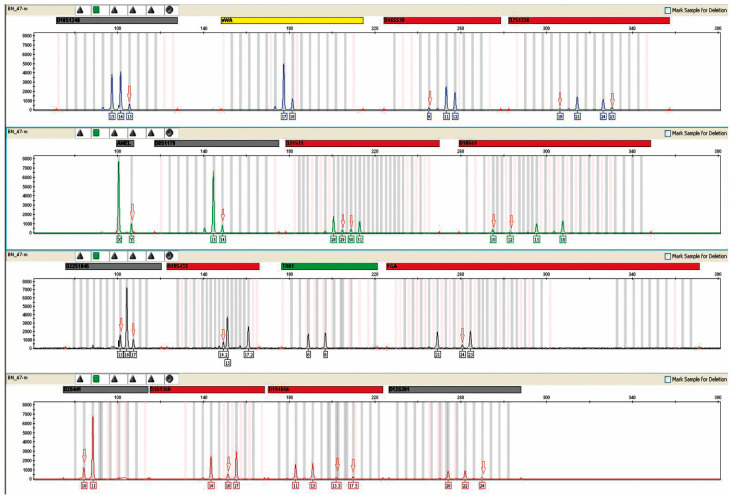
Presence of male DNA in the urine of patient 16. Result obtained after a multiplex PCR reaction performed with an AmpFlSTR^®^ NGM™ PCR Amplification Kit from Applied Biosystems (Waltham, MA, USA). A mixed genetic profile obtained from the urine of the patient under study is shown. A mixture with male DNA is visible, as evidenced by the appearance of the male allele in the amelogenin marker (AMEL). Red arrows indicate alleles derived from donor DNA. The concentration of male DNA in this case was 0.153803 ng/μL. The markers of the systems shown above were labeled with different fluorescent dyes during the course of the multiplex PCR reaction, which translates into different colored peaks corresponding to the alleles.

The presence of male DNA was detected in all urine samples from female kidney recipients. A sample plot is shown in Figure 1. The highest concentration was 6.061994 ng/μL, and the lowest was 0.001752 ng/μL.

#### 2.2.2. Presence of Male DNA in Women’s Blood

**Figure 2:**
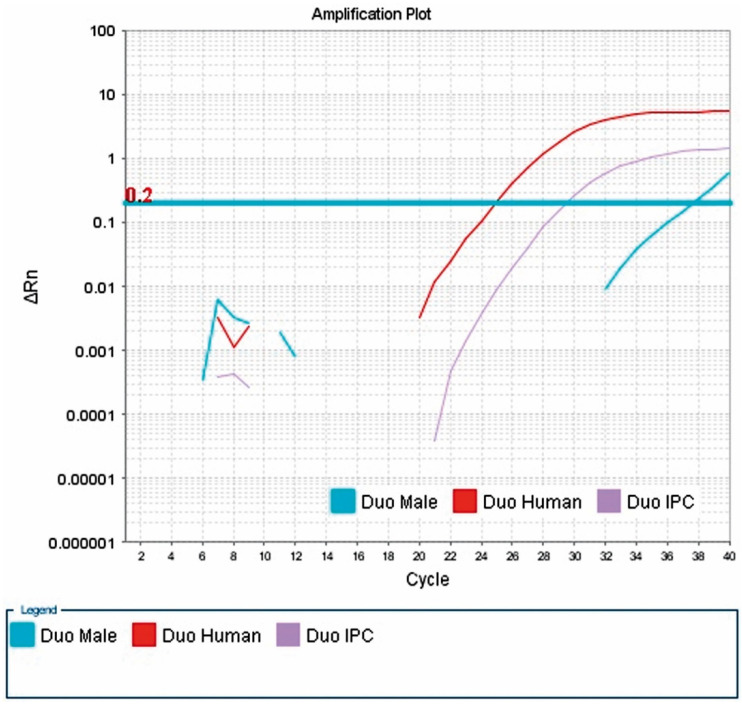
Presence of male DNA in the blood of patient 79. Result of a real-time PCR reaction performed with a Quantifiler^®^ Duo DNA Quantification Kit from Applied Biosystems. The presence of male DNA in the patient’s blood is shown. The Y-chromosome-specific marker used in this kit, multiplying in real time, allows the concentration of male DNA to be determined (Duo Male curve). The X-axis shows successive cycles of the PCR reaction and the Y-axis shows ΔRn, which determines the magnitude of the signal generated by a given set of PCR reaction components. Three curves can be seen: (1) Duo Male (amplification curve of the marker for the SRY gene, located on chromosome Y at position 11.3, for which the probe was labeled with FAM™ dye); (2) Duo Human (amplification curve of the marker for human DNA, i.e., (2) Duo Human (amplification curve of a marker for human DNA, i.e., the 140 bp RPPH1 gene located on the long arm of chromosome 14 at position 11.2, for which the probe was labeled with VIC dye); (3) Duo IPC (amplification curve for the internal IPC standard of 130 bp, for which the probe was labeled with NED™ dye). The threshold line is shown in blue. It is placed above the non-specific deviation ΔRn, within the exponential increase. The copy threshold (CT) number, which is the cycle number at which the amplification curve crosses the threshold line (detectable increase in fluorescence), was determined for the samples.

The presence of male DNA was detected in 13/56 blood samples from female kidney recipients. The highest concentration was 0.037444 ng/μL, and the lowest was 0.00173 ng/μL. The sample result is shown in Figure 2.

### 2.3. Microchimerism in Blood and Urine of Male Kidney Recipients from Female Donors

**Figure 3:**
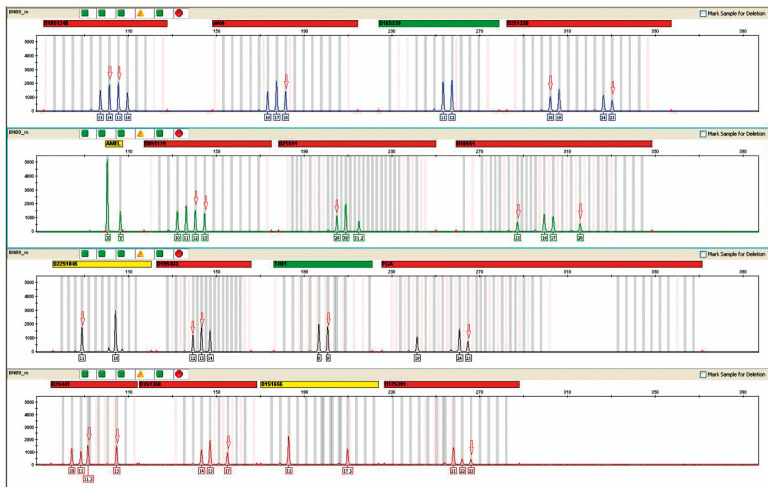
Microchimerism in the urine of patient #74, a recipient of a female donor kidney. The result was obtained after the multiplex PCR reaction performed in the above figure using an AmpFlSTR^®^ NGM™ PCR Amplification Kit from Applied Biosystems. The mixed genetic profile obtained from the urine of the study patient is shown. Red arrows indicate alleles derived from donor DNA. The percentage of female DNA was 80–90%.

The presence of female DNA was found in 31/44 urine samples from male recipients. The highest concentration was 80–90%, and the lowest was 20–30% (Figure 3).

**Figure 4:**
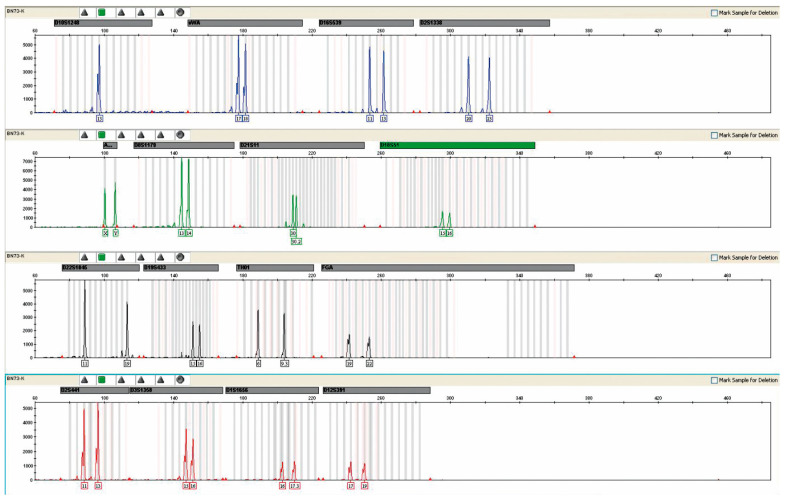
The result of the genetic profile obtained from the blood of patient No. 42, without the presence of foreign DNA. Result obtained after a multiplex PCR reaction.

In men’s blood, the presence of female DNA was not detected in any case by the available test methodology. A representative example of the analysis is shown in Figure 4.

### 2.4. Associations between Microchimerism Present in Blood and Urine of Kidney Recipients and Selected Parameters

Microchimerism in the urine of female recipients correlated positively with urea (Rs = 0.45; *p* = 5.84 × 10^−4^) and K^+^ ions (Rs = 0.29; *p* = 0.03), while microchimerism in the blood of female recipients also correlated positively with urea (Rs = 0. 28; *p* = 0.04) and cystatin C (Rs = 0.31; *p* = 0.02), the number of CD34+CD133+ cells (Rs = 0.26; *p* = 0.06; a statistical tendency) and the number of incompatible HLA alleles (Rs = 0.42; *p* = 0.01). Detailed data are shown in Table 2.

If there was a history of DGF, there were higher concentrations of donor DNA in the urine of both female recipients and male recipients (*p* = 0.041 and *p* = 0.099) than that in recipients with IGF. Data are presented in Table 3.

### 2.5. Comparison of Microchimerism Levels in Women with Male Offspring and Microchimerism in Women without Male Offspring

**Figure 5:**
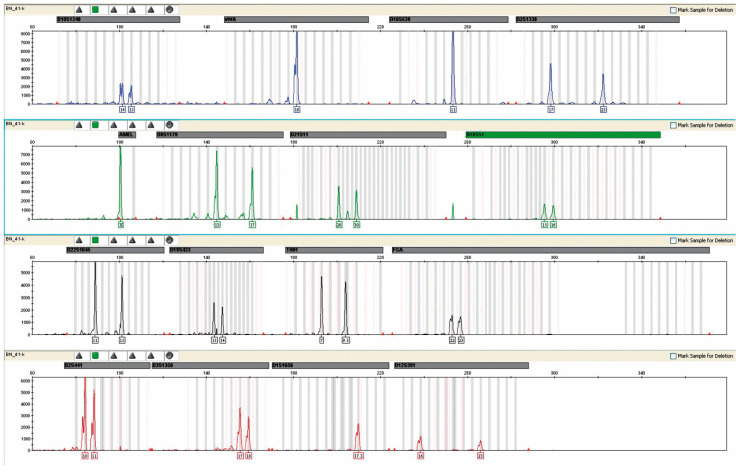
Assessment of maternal–fetal chimerism exemplified by the female profile in one case of disputed paternity showing the maternal profile without any contribution of fetal (male) DNA.

The study found that having a male offspring did not affect the level of microchimerism in female recipients. In addition, to exclude the possible influence of the presence of maternal–fetal microchimerism on the results obtained, 651 women whose blood was used for paternity testing were analyzed as a control group. The study followed an identical methodology (PCR-STR) (Figure 5). No maternal–fetal microchimerism was detected in any of the samples tested. The data are presented in Table 4.

### 2.6. Comparison of Microchimerism Levels in Recipients after a History of Red Cell Concentrate Transfusions and Recipients without Transfusions

A history of previous blood transfusions had no effect on microchimerism levels (Table 5, Figure 5).

## 3. Discussion

A transplanted kidney functioning in the recipient’s body is undoubtedly a phenomenon of modern transplant medicine. Unfortunately, the process of chronic rejection, which often leads to organ loss, remains a problem. The search is still on for markers that herald this process and give a picture of its dynamic changes. In our study involving a group of 100 patients who underwent kidney transplantation at different time points (from 19 days to more than 10 years), quantitative levels of donor DNA were measured in the blood and urine of the recipients. The function of the transplanted kidney was assessed using cystatin C. In addition, the study analyzed the immune status of the recipients by assessing selected immunocompetent cells, such as CD4+ CD25+ regulatory lymphocytes, CD8+ and CD4+ T cells, NK cells, B lymphocytes and monocytes, and their association with the presence of microchimerism. Cystatin C levels in the study group were measured during routine outpatient follow-up, during simultaneous blood and urine sampling for microchimerism and immunocompetent cell analysis. The key parameter analyzed in the study was the level of post-transplant microchimerism in the blood and urine of the recipients. The study required material from patients who had received an organ from a donor of the opposite sex. The selected methods of microchimerism analysis, which are standard in forensic medicine, allowed the differentiation of the study material of different sexes. Using the same tools for single-sex transplants would not have been as conclusive.

The presence of male DNA was detected in all urine samples from female recipients and in 13/56 blood samples from these patients. The positive results for urine were predictable because the transplanted kidney is an organ that is also genetically foreign to the recipient. The urine must therefore contain fragments of the shed epithelium and, therefore, the donor’s cells and DNA. The presence of male DNA in the blood of female patients is a phenomenon similar to that in urine, but much less intense and less detectable. The presence of donor DNA in the recipient’s bloodstream is associated with the ‘scavenging’ of cells from the transplanted organ as the blood flows through the capillaries of the kidney. In women in whom no microchimerism was detected in the blood, its presence cannot be ruled out, but due to its very low level it could not be detected with the techniques used. The presence of female DNA was detected in 31/34 male urine samples. The presence of female DNA was not detected in any male blood sample using the methodology used. In the case of urine, the results obtained can be explained via analogy with those obtained for female recipients. On the other hand, the presence of female DNA in male blood using the molecular techniques used did not give the desired results due to their imperfection. This does not mean that the phenomenon of microchimerism does not exist; it is due to the limitations of the research method and its inability to only be applied to female genetic material.

Many researchers believe that a weakness in the use of microchimerism as a marker of the immune response to a transplanted organ is the described phenomenon of maternal–fetal or embryonic microchimerism. Fetal cells pass through the placenta into the mother’s bloodstream during pregnancy and can eventually take up residence in many tissues [11]. Each pregnancy is a source of fetal cells that migrate into the mother’s body, with multiple pregnancies being the source of a many times larger pool of fetal cells due to the greater placental flow compared with single pregnancies [12,13,14]. On the other hand, there is a bidirectional transfer of fetal and maternal cells during pregnancy, so maternal cells can also be found in the adult offspring [15,16]. Fetal microchimerism is detected in the mother’s body even decades after pregnancy [17,18,19]. Studies suggest that the occurrence of this phenomenon in both mother and child can trigger the development of many autoimmune diseases [20,21]. The presence of maternal–fetal microchimerism and its impact on the development of autoimmune disease remains unclear. It may contribute to the hyperreactivity of the immune system, but it is not known whether it is itself a consequence of autoimmune disease [11].

The donor DNA in the recipient’s blood and urine, determined in this study using PCR-STR methodology, was free of a ‘background’ of maternal–fetal microchimerism. To confirm this, the results of 651 women whose blood was used for paternity testing and who were also mothers of a male offspring were analyzed as a control group. The presence of maternal–fetal microchimerism was not detected in any of the samples tested. In addition, microchimerism levels were compared between women with a male offspring (21/56) and those without a male offspring (35/56). The study clearly showed that the presence of a male offspring had no effect on the microchimerism levels measured in the blood and urine of the recipient women.

Microchimerism also occurs in the body as a result of transfusions of blood products [22]. The sensitive STR method allows detection of 1% of donor cells after transfusion. This raises the question of how long donor cells remain in the recipient’s body after transfusion. A study by Lee et al. found that up to 99.9% of leukocytes are cleared within two days of transfusion, with the cell pool increasing over the course of a week before being completely cleared [23]. According to Busch’s study, no donor lymphocytes were found in the recipient 14 days after transfusion. It is generally believed that donor leukocytes have the ability to expand in the recipient’s body, but their survival is usually short and does not exceed one week [24]. In the study group, 19/100 patients had received a blood transfusion more than three months before the study. Patients with a history of PRBC transfusion were compared with a group of patients without transfusion, taking into account microchimerism levels in blood and urine. No statistically significant differences were found between the two groups. By excluding the presence of maternal microchimerism in female recipients and post-transfusion microchimerism in recipients with a history of transfusions, post-transplant microchimerism was objectively determined in the blood and urine of recipient patients.

Statistical analysis of the results obtained in our study showed a clear positive correlation between cystatin C, urea concentrations and the level of microchimerism in the blood of female recipients, but not males. Such correlations were, however, not found in urine. On the other hand, microchimerism in urine correlated positively with urea and potassium concentrations. Among these variables, cystatin is the strongest one linked to graft function [25]. However, others, like urea [26] and potassium [27], might be indirectly linked to renal failure as well [28]. These results might indicate that deterioration of transplanted kidney function is associated with an increase in post-transplant microchimerism levels. Notably, one should remember that the correlations were only found in a small subgroup of samples analyzed (13/56 female recipients) and need further research to prove their validity.

In our study, a positive correlation was also observed between the degree of microchimerism in female blood and the degree of HLA incompatibility between donor and recipient. This phenomenon is theoretically consistent with existing immunological knowledge stating that HLA matching matters in organ transplantation procedures [29,30,31]. The degree of HLA incompatibility between donor and recipient is one of the main factors determining the immunogenicity of an allograft, especially in the context of direct presentation. A higher number of incompatible antigens in the donor results in a more intense response from the recipient’s immune system. This should be associated with a greater recruitment of CD4+ cells with a helper lymphocyte phenotype, followed by induction of CD8+ cytotoxic lymphocyte function. In this context, microchimerism could result from the release of DNA from transplanted cells following cytotoxic reactions.

In our study, we also compared urine microchimerism in patients with IGF and patients with DGF. A history of DGF was associated with higher levels of donor DNA in the urine of female recipients only. In case of men, a statistical tendency was found. We assume that a higher number of tested patients would definitely explain whether such linkage is present. Gender differences impact aspects of kidney care [32]; however, it does not seem possible that it also applies to the detection of DGF, thus its link with microchimerism. Further studies on this aspect should be taken. Nevertheless, the most common cause of DGF is acute renal tubular necrosis. The most common factors for its occurrence are prolonged cold ischemia time, older age and recipient hypotension [33,34,35,36,37,38,39]. In their study, Bai et al. showed a positive correlation between microchimerism levels and prolonged cold ischemia time, the most common cause of DGF [40]. The cold ischemia phenomenon is associated with the reperfusion process, during which a variety of substances are rapidly washed out of the transplanted organ, including DNA and large amounts of proteins, including antigens recognized by Toll-like receptors (TLRs) present on both transplanted kidney cells and donor and recipient APCs. Reperfusion itself, together with the subsequent local induction of the inflammatory response and the subsequent release of APC-mediated acquired response mechanisms, is likely to account for the association of cold ischemia time with increased microchimerism in the context of DGF. These data suggest that a higher degree of HLA incompatibility between donor and recipient or a history of DGF leads to an increase in microchimerism in the recipient. However, a full explanation of the relationship between the degree of HLA incompatibility and the degree of microchimerism requires further studies in a more homogeneous group of recipients.

The current literature is conflicting on the relationship between the renal rejection process and microchimerism. Some studies suggest a positive role for microchimerism in the recipient, suggesting that it contributes to immune tolerance. Pujal et al. conducted a study in a group of 84 kidney transplant patients at 2, 6, 12 and 18 months after surgery. They found its presence in 56.2% of patients at 2 months and 30.1% at 12 months after surgery. The authors found a positive correlation between donor DNA and a lower incidence of acute rejection [41]. A study by El-Ansary of 20 patients who underwent living donor kidney transplantation showed that higher levels of donor cell microchimerism were associated with better kidney function up to 1 month after surgery and predicted improvement in kidney function at 1 and 3 years of follow-up. The authors concluded that donor cell microchimerism may contribute to a lower immune response and transplant acceptance, although this requires further research and a larger group of patients [42]. Similar results were found by Villari et al. in a 2020 study of 12 female kidney transplant recipients [43]. In the literature, there are also papers describing the occurrence of microchimerism after transplantation, in which it was not found to influence the rejection process of the transplanted organ, as in the studies by Fourtounas et al. and Suberbielle et al. [44,45].

In contrast, Lo et al. investigated the presence of donor DNA in the serum of recipients of 8 livers and 28 kidneys and demonstrated the presence of microchimerism in all liver recipients and in 82% of kidney recipients. They suggested that the source of microchimerism could be cells from the transplanted organ that had undergone apoptosis or ‘homing’ of donor hematopoietic cells, particularly in the liver. The researchers concluded that DNA quantification could be a marker of rejection, but it could also be triggered by infections (e.g., CMV), vascular or biliary complications or cancer developing in the transplanted organ [46]. The concept of a negative impact of donor microchimerism on the function of the transplanted kidney observed in the present study is also supported by reports from other researchers. In 2012, Curcio et al. published the results of a 2-year follow-up of 54 kidney transplant patients. The authors analyzed the recipients’ blood microchimerism levels using quantitative real-time PCR for HLA-DRB1. They correlated the results with the function of the transplanted organ. After 2 years, 38 patients had stable kidney function, while 16 patients showed clear signs of dysfunction. Donor DNA was detected in the serum of 75% of the patients with renal dysfunction. In patients with normal renal function, donor DNA was detected in only 11%. At the end of 2 years of follow-up, 18.1% of patients who had lost a kidney had a high level of microchimerism compared with 7.4% of patients who had this titer [47]. Zhang et al. hypothesized that donor DNA may be present in the recipient’s cell-free urine and that its levels may be associated with rejection of the transplanted kidney. They conducted a study of female recipients of a male donor kidney. The SRY gene on the Y chromosome and beta-globin were used as markers for male DNA. Real-time PCR was used for analysis. The SRY sequence was found in the urine of 82% of female recipients. Patients with acute rejection had a dramatic increase in β-globin DNA levels, which decreased rapidly after the immunosuppressive treatment was changed [48].

Schlitt et al. described the case of a liver transplant patient who developed acute rejection of the organ eight years after the procedure, and therefore underwent another liver transplant. Histopathology of the removed liver showed chronic rejection in addition to severe acute rejection. Before the second transplant, the patient received blood, a piece of skin and bone marrow and, during the procedure, lymph nodes from the small intestine. The presence of donor microchimerism from the first liver was then detected in the recipient’s liver, skin, mesenteric nodes and blood using oligonucleotide probes specific for donor and recipient DRB1 alleles. Recipient tissues were then collected 9 weeks after the second transplantation and the presence of the new microchimerism was detected. On this basis, Schlitt et al. concluded that microchimerism remains in constant equilibrium and its presence is not associated with normal function of the transplanted organ [49]. Gadi et al. analyzed the presence of microchimerism in 42 pancreas–kidney recipients and correlated it with the process of acute rejection confirmed via histopathological examination. They assessed microchimerism using real-time PCR. One week after transplantation, they detected donor DNA in 94% of patients. Patients were followed up for 5 years after transplantation. The amount of donor DNA was higher in patients with a history of organ rejection compared with a group of patients without rejection. After pharmacological intervention in the rejection process, the amount of donor DNA decreased significantly. Despite the results obtained, Gadi et al. concluded that the presence of donor DNA in the recipient’s serum did not correspond to the rejection process and cannot be used as a parameter of dysfunction. The presence of donor DNA can be part of an algorithm to determine the onset of rejection of the transplanted organ. In conclusion, the researchers found that assessing the level of donor DNA in the recipient’s body is effective in managing immunosuppression [50]. Summarizing the above research and as the highest proof in evidence-based medicine, a meta-analysis by Knight et al. [51] clearly proved that the presence of a donor-specific, cell-free DNA negatively correlated with a solid transplant function and might serve as marker of a graft injury. Our study has some limitations. The major one is that due to financial constraints, microchimerism testing was performed only once in each patient with different time post-surgery. This for sure influenced the results, as the time frame between time from transplantation (days) varied significantly. However, as elegantly demonstrated, a rapid fall in cfDNA content of a donor by approximately 10 days post-transplantation occurred and then tended to stabilize; of course, this had some variation for living vs. deceased donors and different solid organs transplanted [51]. Also, the study required material from patients who had received an organ from a donor of the opposite sex. The selected methods of microchimerism analysis, which are standard in forensic medicine, allowed the differentiation of opposite-sex study material. Applying the same tools to unisex transplants would not have been as conclusive. Also, the results of correlation analyses refer to a relatively small subgroup of patients (13/56 female recipients); thus, the data we obtained needs to be verified in further research. Indeed, a correlation (found in the present study) does not exactly mean causation.

Both positive and negative effects of post-transplant microchimerism on transplanted kidney function have been reported in the literature. The discrepancy in results is due to the lack of standardized research methods. Also, studies evaluating microchimerism used different fractions of blood to obtain DNA, for instance separated mononuclear cells or whole peripheral blood (similarly to our approach). This might result in different fractions of DNA isolated and, thus, analyzed of intact cell origin DNA vs. cell-free DNA. In summary, the results obtained by researchers, although so different, are not necessarily mutually exclusive. It is very likely that the presence of traces of donor DNA in the recipient of a transplanted organ promotes the phenomenon of immunological tolerance. Certain external conditions, such as a history of severe infections (especially CMV) and cytotoxic effects of drugs, disrupt immune homeostasis and the function of the transplanted kidney deteriorates. In this situation, an increase in microchimerism levels should be a signal to modify the current immunosuppressive treatment. Standardization of testing methods for donor DNA traces may contribute to the recognition of microchimerism testing as one of the markers of organ function risk.

## 4. Materials and Methods

### 4.1. Study Group

After approval by the Bioethics Committee of the Medical University of Pomerania in Szczecin (Resolution No. KB-0080/128/09), there were 100 kidney recipients aged 20–78 years (mean 49.91 years) from the Transplantation Outpatient Clinic of the Department of Nephrology, Transplantation and Internal Medicine, Independent Public Clinical Hospital no. 2 of the Pomeranian Medical University in Szczecin and the Outpatient Transplant Clinic of the Department of Nephrology and Kidney Transplantation with Dialysis Unit of the Independent Regional Public Hospital in Szczecin. All patients included in the study received detailed information about the study and gave written informed consent for the study to be conducted. All transplanted organs were from deceased donors. To be included in the study, patients had to have received a kidney from a donor of the opposite sex.

All recipients were further analyzed for body mass index (BMI), blood group, duration (in months) and type of dialysis, cause of renal failure, previous red blood cell (RBC) transfusions, occurrence of delayed graft function (DGF) after transplantation, occurrence of histopathologically confirmed acute rejection, postoperative immunosuppression (primary and current), average daily urine output (in liters), biochemical tests, baseline blood count and number of incompatible alleles in the HLA system between donor and recipient. All analyses were carried out according to the rules of the clinical laboratory.

HLA compatibility between recipients and their donors was calculated according to the current recommendations of the Polish allocation system. Transplants without mismatches were scored as follows: 2 points for each absence of mismatch at A loci, 5 points at B loci and 10 points at DR loci.

The study used the results of the genetic profiles of 651 women from paternity analyses to determine maternal–fetal microchimerism. The presence of donor DNA (intact) in the recipient’s blood and urine (post-transplant microchimerism) was analyzed by quantifying the ratio of donor to recipient DNA content.

### 4.2. Determination of Cystatin C

An immunonephelometric method was used for the determination of cystatin C. The analysis was performed on a Siemens BN ProSpec nephelometer. A Siemens N La-tex Cystatin C diagnostic test was used for the determination. The procedure was performed according to the manufacturer’s protocol.

### 4.3. Determination of Donor DNA in Blood and Urine of the Recipient

#### 4.3.1. Cell-Free DNA Isolation

DNA was isolated from two types of biological material: whole peripheral blood and urine. A commercially available PrepFiler Forensic DNA Extraction Kit was used for isolation. The biological material was properly prepared prior to isolation. Urine was centrifuged and the precipitate collected on a sterile swab. Blood was applied to an FTA card, from which small sections (approximately 25 mm^2^) were cut after drying. Before starting the process, the magnetic beads provided were incubated at 37 °C for 10 min and then vortexed. Isolation was performed according to the manufacturer’s protocol [52].

#### 4.3.2. DNA Amplification

The PCR reaction was performed on a GeneAmp^®^ PCR System 9700 thermocycler [53], and an AmpFlSTR^®^ NGM™ PCR Amplification Kit from Applied Biosystems [54] was used for the multiplex PCR reaction, amplifying 15 loci from 34 short tandem repeats (STRs): D10S1248, vWA, D16S539, D2S1338, D8S1179, D21S11, D18S51, D22S1045, D19S433, TH01, FGA, D2S441, D3S1358, D1S1656, D12S391 and a marker located on the sex chromosomes—amelogenin. The reaction mixture consisted of (per 25 µL sample)

- 10 µL AmpFlSTR^®^ NGM™ Master Mix;
- 5 µL AmpFlSTR^®^ NGM™ Primer Set;
- 10 µL test DNA.

The positive control consisted of 10 µL AmpFlSTR^®^ NGM™ Master Mix, 5 µL AmpFlSTR^®^ NGM™ Primer Set and 10 µL control DNA, and the negative control consisted of 10 µL AmpFlSTR^®^ NGM™ Master Mix, 5 µL AmpFlSTR^®^ NGM™ Primer Set and 10 µL deionized water. The amplification program followed the manufacturer’s recommendations [55].

#### 4.3.3. Capillary Electrophoresis

Samples were analyzed using a four capillary Applied Biosystems 3130 Genetic Analyzer [56]. GeneMapper^®^ ID Software 3.1 and 3.2 was used to process the results [57]. After amplification, the DNA was separated electrophoretically in a 96-well plate. To create the reaction mixture, the following were used:

- 0.3 µL GS 500 LIZ^®^ Size Standard;
- 8.7 µL Hi-Di™ Formamide;
- 1 µL of PCR product/allelic ladder.

The composition of a negative control included:

- 0.3 µL GS 500 LIZ^®^ Size Standard;
- 8.7 µL Hi-Di™ Formamide [58].

#### 4.3.4. Assessment of DNA Concentration

The microchimerism observed after the multiplex PCR reaction was further confirmed by assessing the concentration of male DNA in samples from recipient patients. This reaction, in addition to confirming the presence of foreign DNA, allowed a quantitative assessment of the microchimerism phenomenon.

The analysis was based on the real-time polymerase chain reaction method (real-time PCR). The reaction was performed using a Quantifiler^®^ Duo DNA Quantification Kit from Applied Biosystems [59]. It allowed both the detection of total nuclear DNA concentration and the measurement of male DNA concentration. As an internal standard, a series of 8 dilutions of the concentration standards were prepared according to the manufacturer’s recommendations, with the following concentrations: 50, 16.7, 5.56, 1.85, 0.62, 0.21, 0.068, 0.023 ng/µL. The reaction mixture (for one sample) consisted of:

- 10.5 µL Quantifiler Duo Primer Mix;
- 12.5 µL Quantifiler Duo PCR Reaction Mix;
- 2 µL of test DNA.

A negative control was also performed. Samples were added to a 96-well plate according to the manufacturer’s recommended scheme and the reaction was performed on an Applied Biosystems 7500 RealTime PCR System machine [60]. Results were analyzed using HID RealTime PCR-Analysis Software v. 1.1 [61].

#### 4.3.5. Quantitative Evaluation of Female DNA in The Urine of Male Recipients

Genetic profiles obtained after multiplex PCR reactions using an AmpFlSTR^®^ NGM™ PCR Amplification Kit from Applied Biosystems were analyzed. Those with a mixed profile indicative of foreign DNA were evaluated. Evaluation was based on peak height measurements for the donor and recipient alleles using GeneMapper^®^ software 3.1 and 3.2 [57] based on the ratio of donor to recipient peak heights read from electropherograms measured with relative fluorescence units (RFUs) in male patients who received a transplant from a female donor. Peak heights were used to estimate the percentage of donor DNA content in the recipient.

### 4.4. Immunocompetent and Stem Cell Research

Analysis of immunocompetent and stem cells was performed using flow cytometry according to the methodology described in the author’s previously published articles [62,63,64].

### 4.5. Statistical Analysis

Spearman’s rank correlation coefficient (Rs) was used to assess associations between tested variables of continuous type. Correlations with *p* < 0.05 were considered statistically significant. The *t*-test was used to assess the association between qualitative variables and the parameters tested. Calculations were carried out using Statistica 10.

## 5. Conclusions

- Increased levels of post-transplant microchimerism in female graft recipients might correlate with deterioration of transplanted kidney function as expressed by cystatin C levels and HLA match and indirectly by potassium and urea.
- A history of DGF leads to increased levels of microchimerism.
- The degree of microchimerism in recipients might be considered as a new predictive/prognostic marker in renal transplantation.

## Figures and Tables

**Table 1:** Clinical characteristics of studied patients.

Parameter (Unit)	n	Mean	SD	Median	Minimum	Maximum
Age of recipient (years)	100	49.91	12.67	52	20	78
Age of donor (years)	84	44.21	12.81	46	16	66
Time from transplantation (months)	100	60.43	27.69	63.89	0.62	127.02
BMI (kg/m^2^)	98	26.41	4.14	26.1	18	44
Duration of dialysis (months)	98	22.37	25.90	14	0	192
Mean urine 24 h Volume (L/d)	89	2.64	0.61	2.5	1.5	4
Urea (mg/dL)	98	38.91	24.24	34.05	10.5	134.3
Creatinine (mg/dL)	100	1.42	0.66	1.23	0.7	4.02
Glucose (mg/dL)	98	100.72	23.76	95	52	218
Uric acid (mg/dL)	99	6.99	1.68	6.9	2.9	13.5
ALAT (IU/L)	98	27.70	30.50	20	10	252
Na^+^ (mmol/L)	100	139.55	2.38	139.9	132	145.6
K^+^ (mmol/L)	100	4.32	0.54	4.23	2.61	5.8
Mg^2+^ (mmol/L)	98	0.76	0.10	0.75	0.5	1.03
Mismatched HLA alleles	77	2.94	0.007	3	0	6
eGFRMDRD (mL/min/1.73 m^2^)	100	56.63	20.56	55.63	13.21	114.32
Cystatin C (mg/L)	98	1.49	0.61	1.285	0.748	3.86
Microchimerism in female blood (ng/μL)	56	0.002593	0.006866	0	0	0.037444
Microchimerism in female urine (ng/μL)	56	1.36	1.49	0.807969	0.001752	6.061994
Microchimerism in male urine (ng/μL)	44	32.61	26.47	35	0	85

**Table 2:** Correlations of microchimerism with some parameters.

Correlated Parameters		Microchimerism In Female Urine	Microchimerism In Female Blood	Microchimerism In Male Urine
Urea	Rs	0.45	0.28	0.11
	*p*	**0.0006**	**0.04**	0.48
K^+^ ions	Rs	0.29	0.17	0.00
	*p*	**0.03**	0.21	0.98
Uric acid	Rs	−0.01	0.23	−0.23
	*p*	0.95	0.09	0.14
Cystatin C	Rs	0.16	0.31	0.12
	*p*	0.23	**0.02**	0.44
CD34+CD133+	Rs	−0.04	0.26	0.02
	*p*	0.80	0.06	0.88
CD8	Rs	0.06	0.23	−0.23
	*p*	0.67	0.10	0.14
Number of mismatched HLA	Rs	0.23	0.42	0.01
	*p*	0.14	**0.01**	0.97

*p*—statistical significance.

**Table 3:** Comparison of the presence of microchimerism depending on immediate (IGF) or delayed graft function (DGF).

Parameter	IGF		DGF		*p*
	Mean	SD	Mean	SD	
Microchimerism in female urine	1.211	1.403	2.071	1.728	**0.041**
Microchimerism in female blood	0.003	0.007	0.002	0.005	**0.703**
Microchimerism in male urine	31.970	27.270	50.000	15.166	0.099

*p*—statistical significance, SD–standard deviation.

**Table 4:** Comparison of microchimerism depending on having a male offspring.

Parameter	Son (−)		Son (+)		*p*
	Mean	SD	Mean	SD	
Microchimerism in female blood	0.001397	0.004330	0.002789	0.005832	0.2757
Microchimerism in female urine	0.978804	1.011451	1.461379	1.493555	0.4752

*p*—statistical significance, SD—standard deviation.

**Table 5:** Comparison of microchimerism depending on past PRBC transfusions.

Parameter	PRBC (−)		PRBC (+)		*p*
	Mean	SD	Mean	SD	
Microchimerism in female blood	0.001107	0.002558	0.001671	0.005789	0.522927
Microchimerism in female urine	1.218075	1.504353	1.704863	1.653665	0.203137
Microchimerism in male urine	31.52174	25.380504	43.57142	22.860863	0.207457

*p*—statistical significance, SD—standard deviation.

## Data Availability

All data are available upon request from the first author.
